# P-194. Feasibility and effectiveness of standard World Health Organization-recommended treatment in a community setting for Buruli ulcer in Togo: a prospective pilot study

**DOI:** 10.1093/ofid/ofaf695.417

**Published:** 2026-01-11

**Authors:** Richard R Lueking, Stephanie Kirk, Kpalma Duga Bakpatina-Batako, Susan E Dorman

**Affiliations:** Medical University of South Carolina, Charleston, SC; Medical University of South Carolina, Charleston, SC; Floreal Medical Clinic, Lome, Maritime, Togo; Medical University of South Carolina, Charleston, SC

## Abstract

**Background:**

*Mycobacterium ulcerans* is the etiologic agent of Buruli ulcer (BU), an infectious disease characterized by progressive cutaneous ulcerations that can lead to disability, loss of economic productivity, and stigma. Current World Health Organization (WHO) guidelines recommend treatment initiation in the hospital setting or coordinated in a decentralized health center. This prospective pilot study sought to evaluate feasibility and effectiveness of a community-care-based model of BU care.

**Figure:**
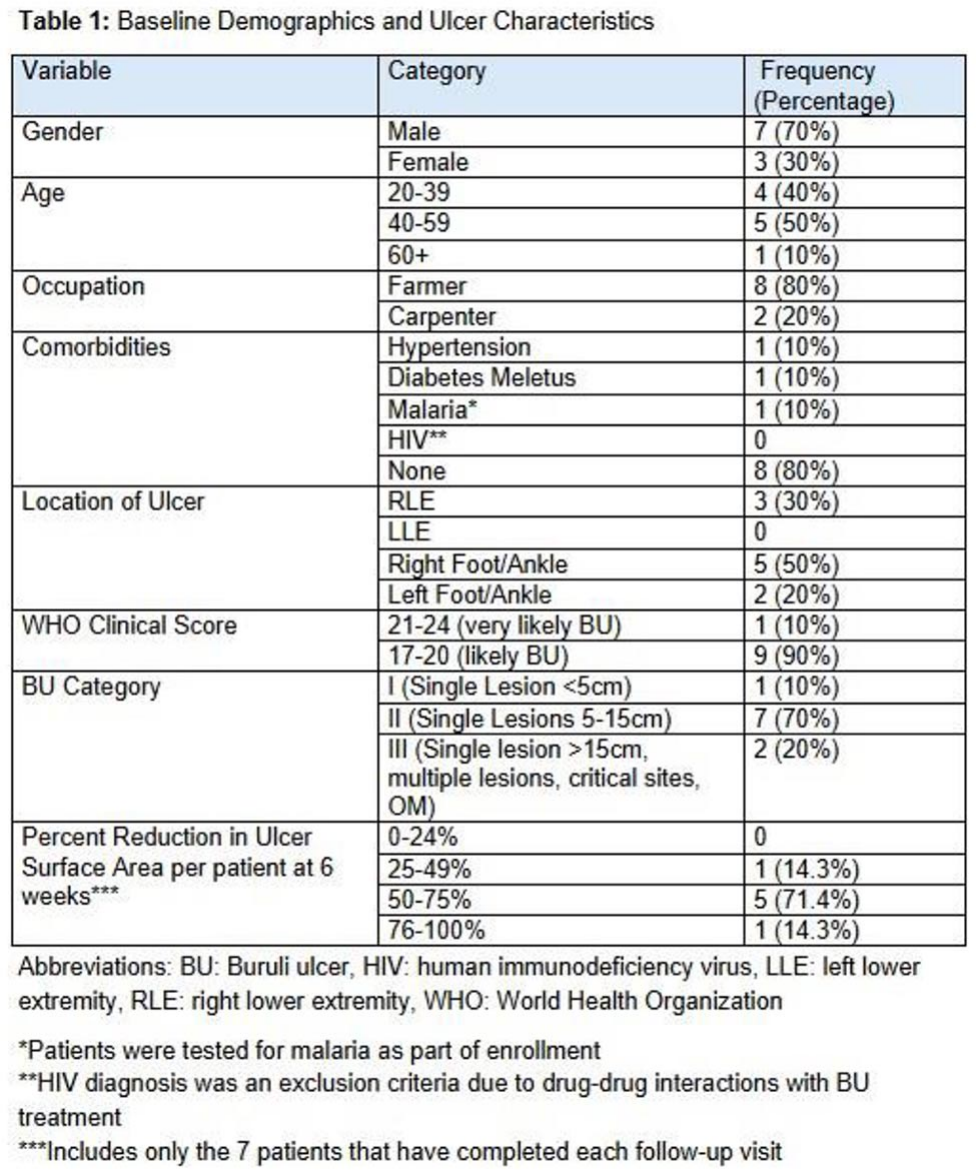

**Figure:**
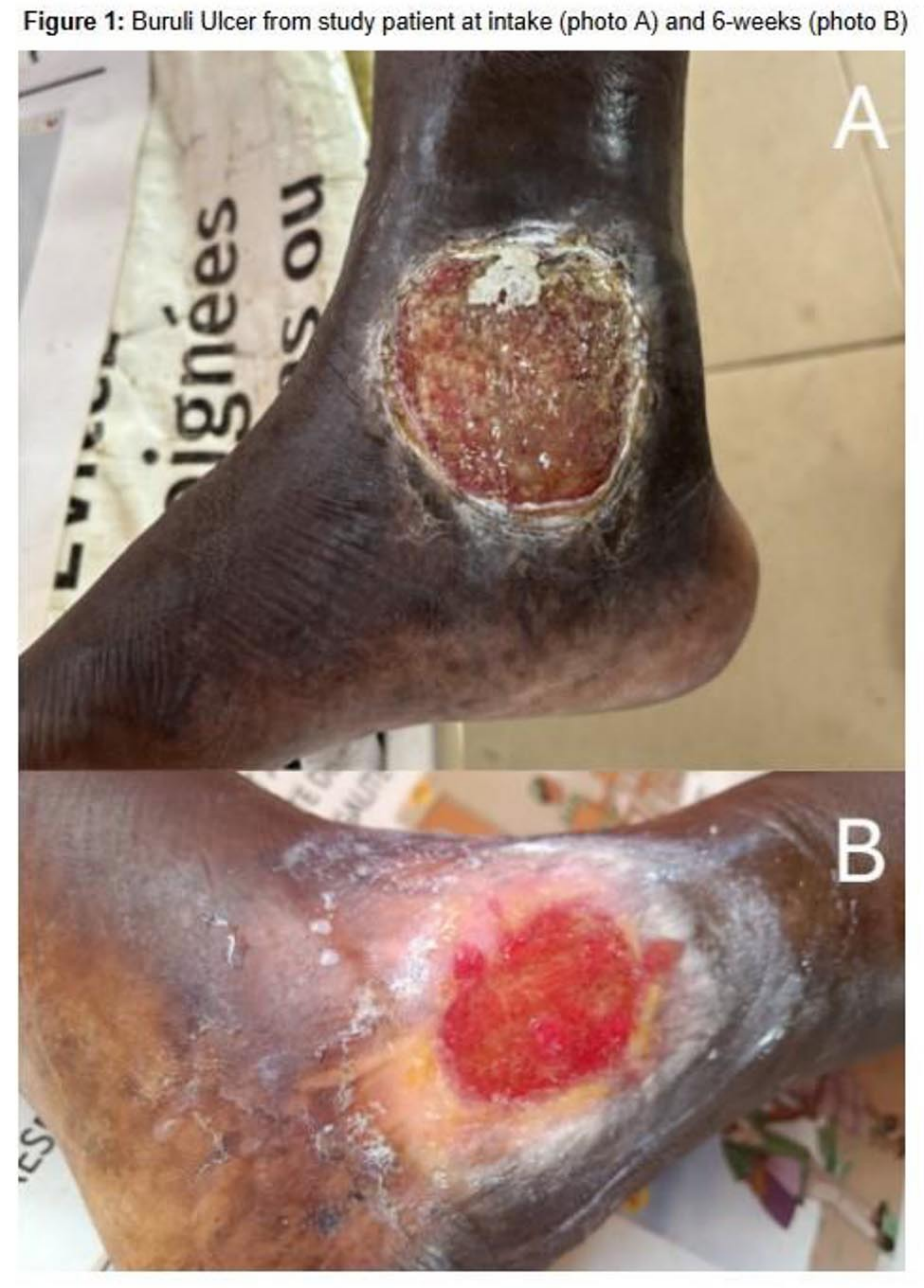

**Methods:**

For this ongoing study, the setting is outpatient general medical clinics in the Plateaux region of Togo, Africa. Consenting adults with cutaneous lesions less than 15cm (WHO Grade I-II or III with multiple small lesions) consistent with BU were enrolled. Participants were provided with standard-of-care, weight-based clarithromycin and rifampin and wound care supplies. The study team provided education on routine wound care that can be done at home. Enrolled participants were visited at home by a community leader every 2 weeks for wound care/dressing education during the 8 weeks of antibiotic treatment. Participants will have home follow-up visits at months 3 and 6 for outcome assessments. At each visit, ulcer measurements and photographs are taken, and standardized checklists are completed to assess treatment adherence, medication side effects, and paradoxical reactions.

**Figure:**
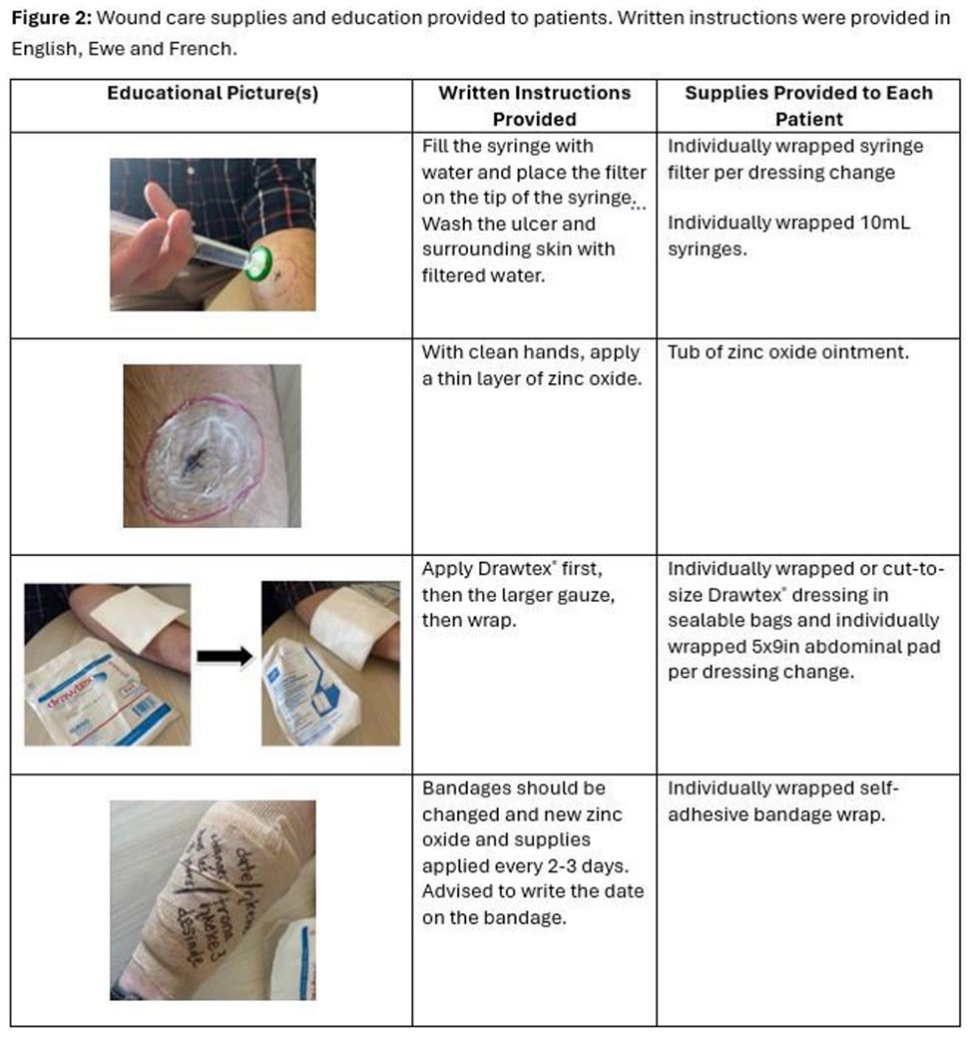

**Results:**

A total of 16 patients were screened, and 10 patients were enrolled and currently undergoing home-based antibiotic treatment and wound care. Three (33%) patients have not completed all follow-up visits. Of those who have completed all visits, they report 100% adherence to medications and wound care therapy at home. To date there have been no reported medication side effects, and 1 (10%) patient had findings consistent with a paradoxical reaction. Interim data shows ulcer size improved in all 7 patients that have completed visits, with an average reduction in size (cm3) of 64.7% and complete epithelization in 1 patient at the 6-week mark.

**Conclusion:**

Early results support the feasibility of community-based treatment for individuals with (WHO Grade I-II disease or Grade III with multiple lesions of small size) *Mycobacterium ulcerans* cutaneous disease.

**Disclosures:**

Stephanie Kirk, PharmD, ViiV Healthcare: Grant/Research Support

